# Galectin‐1‐Induced Tumor Associated Macrophages Repress Antitumor Immunity in Hepatocellular Carcinoma Through Recruitment of Tregs

**DOI:** 10.1002/advs.202408788

**Published:** 2025-01-24

**Authors:** Xizhi Yu, Junjie Qian, Limin Ding, Caixu Pan, Xi Liu, Qinchuan Wu, Shuai Wang, Jianpeng Liu, Mingge Shang, Rong Su, Danjing Guo, Haiyang Xie, Shengyong Yin, Lin Zhou, Shusen Zheng

**Affiliations:** ^1^ Division of Hepatobiliary and Pancreatic Surgery, Department of Surgery, The First Affiliated Hospital, School of Medicine Zhejiang University Hangzhou 310003 China; ^2^ NHC Key Laboratory of Combined Multi‐organ Transplantation Key Laboratory of Organ Transplantation Zhejiang 310003 China; ^3^ Key Laboratory of the diagnosis and treatment of organ Transplantation, Research Unit of Collaborative Diagnosis and Treatment for Hepatobiliary and Pancreatic Cancer Chinese Academy of Medical Sciences (2019RU019) Hangzhou 310003 China; ^4^ State Key Laboratory for Diagnosis and Treatment of Infectious Diseases National Clinical Research Center for Infectious Diseases Hangzhou 310003 China; ^5^ Department of Hepatobiliary and Pancreatic Surgery, Department of Liver Transplantation Shulan (Hangzhou) Hospital Affiliated to Zhejiang Shuren University Shulan International Medical College Hangzhou 310000 China

**Keywords:** Galectin‐1, hepatocellular carcinoma, immunotherapy, tumor‐associated macrophages, tregs

## Abstract

Tumor‐associated macrophages (TAMs) are commonly considered accomplices in tumorigenesis and tumor development. However, the precise mechanism by which tumor cells prompt TAMs to aid in evading immune surveillance remains to be further investigated. Here, it is elucidated that tumor‐secreted galectin‐1 (Gal1) conferred immunosuppressive properties to TAMs. Specifically, patient specimens and a public database is first used to analyze the clinical relevance of Gal1 in hepatocellular carcinoma (HCC). Then, it is demonstrated that TAMs functioned as a critical mediator in the Gal1‐induced progression of HCC and the establishment of an immunosuppressive tumor microenvironment. Furthermore, RNA‐sequencing determined that Gal1 promoted the upregulation of chemokine (C‐C motif) ligand 20 (CCL20) in TAMs via activating the PI3K/AKT/NF‐κB pathway. Employing an anti‐CCL20 neutralizing antibody and *Foxp3DTR* mice, it is demonstrated that CCR6^+^Foxp3^+^ regulatory T cells (Tregs) recruited by Gal1‐induced TAMs contributed to reduced infiltration and dysfunctional state of CD8^+^ T cells, subsequently facilitating tumor progression. Targeting Gal1 dampened the secretion of CCL20 and inhibits the recruitment of Tregs, thereby activating anti‐tumor immunity and ameliorating anti‐PD‐1 resistance. Together, this findings revealed that Gal1‐induced TAMs recruited Tregs through the CCL20‐CCR6 axis. Inhibition of Gal1 improves the effectiveness of anti‐PD1 therapy, shedding important new light on the combination immunotherapy of HCC.

## Graphical abstract

Gal1‐induced TAMs recruited CD4^+^CD25^+^Foxp3^+^CCR6^+^ Tregs through the CCL20‐CCR6 axis and culminated in impaired anti‐tumor immune responses in HCC.

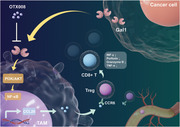

## 1 Introduction

Hepatocellular carcinoma (HCC) is one of the most commonly diagnosed cancers and the third leading cause of death from cancer worldwide.^[^ ^1^ ^]^ Immune checkpoint inhibitors (ICIs) targeting co‐inhibitory molecules, which include cytotoxic T lymphocyte‐associated antigen 4 (CTLA4), programmed cell death‐1 (PD‐1), or programmed cell death‐ligand 1 (PD‐L1), are the first immunotherapy agents to prove effective against HCC.^[^ ^2^ ^]^ However, as the backbone of ICIs, the PD‐1 inhibitors nivolumab and pembrolizumab only produce a 15–20% objective remission rate in HCC.^[^ ^2^, ^3^, ^4^ ^]^ Numerous studies indicated that the suppressive tumor microenvironment (TME) was pivotal in the unfavorable response to ICIs.^[^ ^5^, ^6^, ^7^ ^]^ As the most infiltrated immune cells in the TME, TAMs are a heterogeneous and plastic population regulating the initiation, development, and metastasis of tumors and have been shown to be a reliable target for optimizing the efficacy of immunotherapy.^[^ ^8^, ^9^, ^10^, ^11^ ^]^ Mechanistically, TAMs promote neo‐angiogenesis and cancer cell intravasation into the circulation.^[^ ^12^, ^13^, ^14^ ^]^ Additionally, TAMs also express cell‐surface receptors and secret an array of chemokines, cytokines, and enzymes that suppress the effector function of tumor‐infiltrated lymphocytes directly or indirectly through the induction or recruitment of Tregs.^[^ ^11^, ^14^, ^15^ ^]^ Of note, tumor cells, in turn, recruit new monocytes and facilitate the transformation of macrophages into pro‐tumoral phenotype via a range of mechanisms so that TAMs evolve along with the tumor progression.^[^ ^11^, ^16^, ^17^ ^]^ Consequently, an in‐depth dissection of the crosstalk between tumor cells and TAMs will help us better understand the evolution of the immunoinhibitory TME and uncover suitable therapeutic targets.

Galectin‐1(Gal1), a tandem repeat‐type of 14‐kDa protein, is highly expressed and secreted into the milieu by a spectrum of primary and metastatic tumors. In addition, Gal1 is characterized by two carbohydrate recognition domains that extensively bind to the glycolipid or glycoprotein on the cell surface, thus modulating a cascade of downstream pathways.^[^ ^18^, ^19^ ^]^ Accumulating evidence has shown that Gal1 is involved in the maintenance of tumor immune tolerance and is associated with tumor progression and poor prognosis in patients.^[^ ^20^, ^21^, ^22^ ^]^ A recent study demonstrated that Gal1 also fueled the metastatic progression of head and neck cancer by favoring the CXCL2 expression of cancer cells to facilitate MDSC recruitment.^[^ ^23^ ^]^ However, the role of Gal1 in regulating the tumor immune microenvironment of HCC remains poorly understood. Meanwhile, as a tumor‐secreted immunosuppressive protein, whether Gal1 could be a significant player in endowing TAMs with pro‐tumoral capacity is a great concern. Although there is some suggestion that Gal1 may induce TAMs to express M2 markers,^[^ ^24^, ^25^ ^]^ the specific regulatory functions and the underlying mechanisms remain elusive.

Here, we unraveled that TAMs were involved in the progress of tumor progression and immune escape induced by Gal1, implicating that Gal1 participated in the crosstalk between tumor cells and TAMs in HCC. We demonstrated that Gal1‐induced TAMs activated the CCL20 release via stimulating the PI3K/AKT/NF‐κB pathway, therefore triggering the recruitment of CCR6^+^Foxp3^+^ Tregs that impaired the anti‐tumor response of CD8^+^ T cells. Finally, targeting Gal1 reduced the interplay between TAMs and HCC cells, thwarted the infiltration of Tregs, and culminated in significant augmentation of the anti‐PD1 therapeutic efficacy. Our findings suggested that tumor‐derived Gal1 was a prognostic biomarker, and targeting Gal1 promoted the efficacy of anti‐PD1 therapy, which provided a potential combination strategy against HCC.

## 2 Results

### 2.1 TAMs Play a Crucial Role in the Gal1‐Induced Tumor Progression

**Figure 1:**
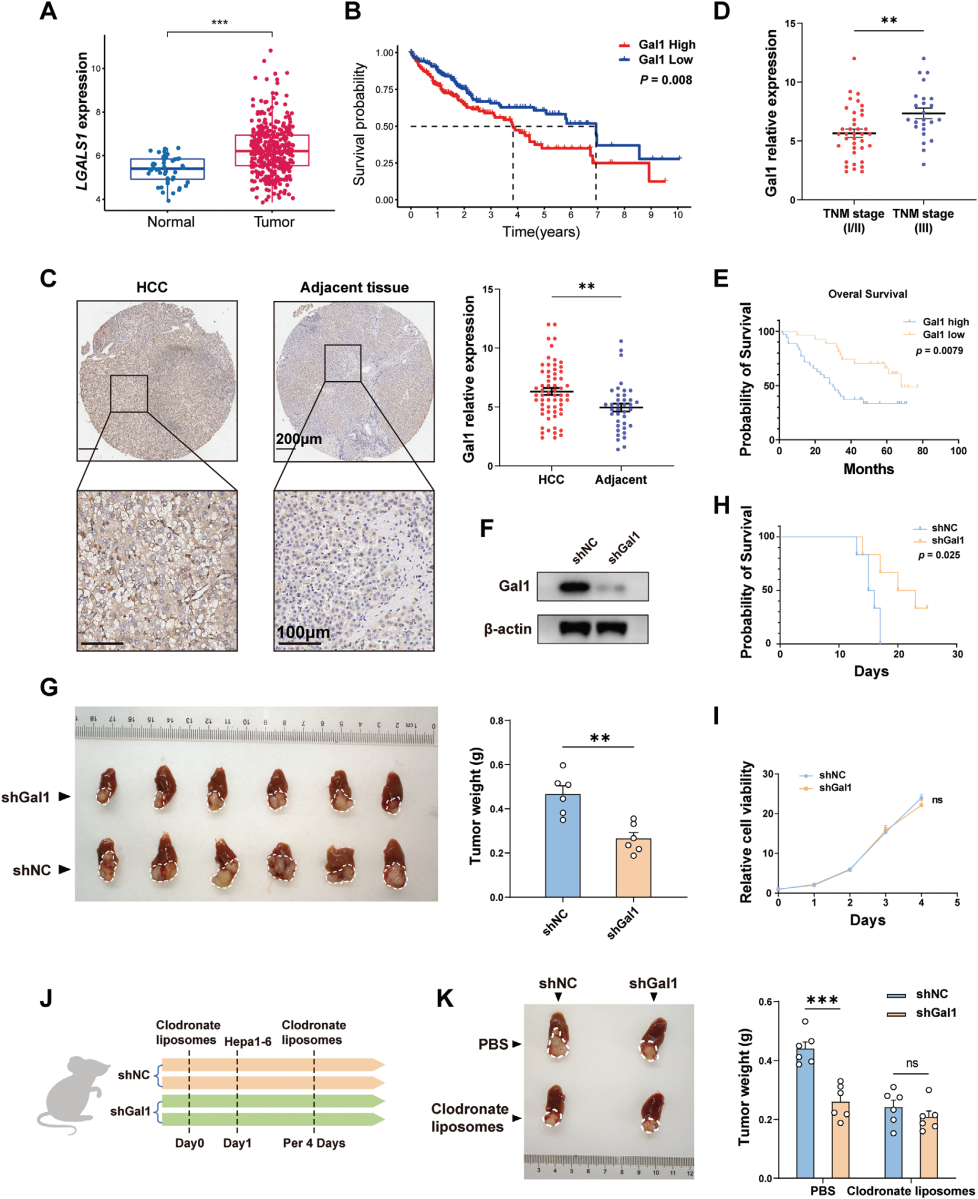
TAMs play a crucial role in the Gal1‐induced tumor progression. A) *LGALS1* expression in normal tissues and tumor tissues of HCC patients based on TCGA database. B) Comparison of overall survival between Gal1 high expression and Gal1 low expression groups in TCGA cohort. C) Representative IHC staining of Gal1 in HCC tissue array (left) and comparison of relative Gal1 expression between normal tissues (n = 38) and tumor tissues (n = 62) according to immunohistochemical staining score (right). D) Comparison of Gal1 expression between patients with early stage (TNM stage I/ II, n = 38) and advanced stage (TNM stage III, n = 24). E) Kaplan‐Meier curve analysis of overall survival of HCC patients with high Gal1 expression (IHC staining score ≥ 6, n = 35) compared with patients with low Gal1 expression (IHC staining score < 6, n = 27). F) Western blot analysis of Gal1 protein expression in shNC and shGal1 hepa1‐6 cells. G) Images of HCC tumor (left) and tumor weight (right) in the orthotopic HCC mouse models (n = 6). H) Comparison of overall survival between shNC and shGal1 orthotopic tumor models (n = 6). I) The proliferation of shNC and shGal1 cells measured by CCK8 assay. J) Flow chart of shNC and shGal1 cell implantation and macrophage depletion. K) Representative image of tumor (left) and tumor weight (right) in the orthotopic HCC mouse models with or without macrophage depletion (n = 6). Data were presented as mean ± SEM. ^*^ *P* < 0.05; ^**^ *P* < 0.01; ^***^ *P* < 0.001; ^****^ *P* < 0.0001.

To assess the potential association between Gal1 and the progression of HCC, we conducted bioinformatic analyses on the HCC patient data from The Cancer Genome Atlas (TCGA) database. The results revealed a higher level of Gal1 expression in HCC compared to adjacent tissues (**Figure** **1**A). Moreover, a high level of Gal1 expression in HCC indicated a poor prognosis (Figure 1B). To further substantiate these findings, an additional analysis was performed on HCC tumor tissues (n = 62) and adjacent noncancerous liver tissues (n = 38) from the First Affiliated Hospital of Zhejiang University School of Medicine using immunohistochemistry (IHC). The results further confirmed the high expression of Gal1 in tumor tissues and a high stage and poor prognosis of HCC patients with Gal1 high expression (Figure 1C–E; Figure S1A–C, Supporting Information). Following this, we constructed stable Gal1 knockdown hepa1‐6 cell lines (Figure 1F) and established liver orthotopic HCC mouse model via injecting Gal1 knockdown (shGal1) or control (shNC) hepa1‐6 cells. We found that Gal1 knockdown significantly impeded tumor growth and extended the survival time of mice bearing tumors (Figure 1G,H). To evaluate the impact of Gal1 knockdown on the cell line, we assessed cell proliferation using the CCK8 assay and found no significant difference between shGal1 and shNC (Figure 1I). Therefore, we propose that the tumor‐promoting effects of Gal1 may be mediated through its impact on the immune system. TAMs are the most abundant tumor‐infiltrating immune cells in HCC, and our previous studies indicated the vital role of TAMs in promoting HCC development through facilitating the formation of an immunosuppressive tumor microenvironment.^[^ ^26^, ^27^ ^]^ To investigate the impact of TAMs in Gal1‐mediated HCC progression, we utilized clodronate liposomes to deplete macrophages (Figure 1J; Figure S2A, Supporting Information). Intriguingly, the absence of macrophages in the liver attenuated the tumor suppressive effect of Gal1 knockdown, indicating that TAMs mediated the tumor‐promoting effect of Gal1 on HCC (Figure 1K).

### 2.2 TAMs are Critical Mediators in the Establishment of Immunosuppressive TME Induced by Gal1

**Figure 2:**
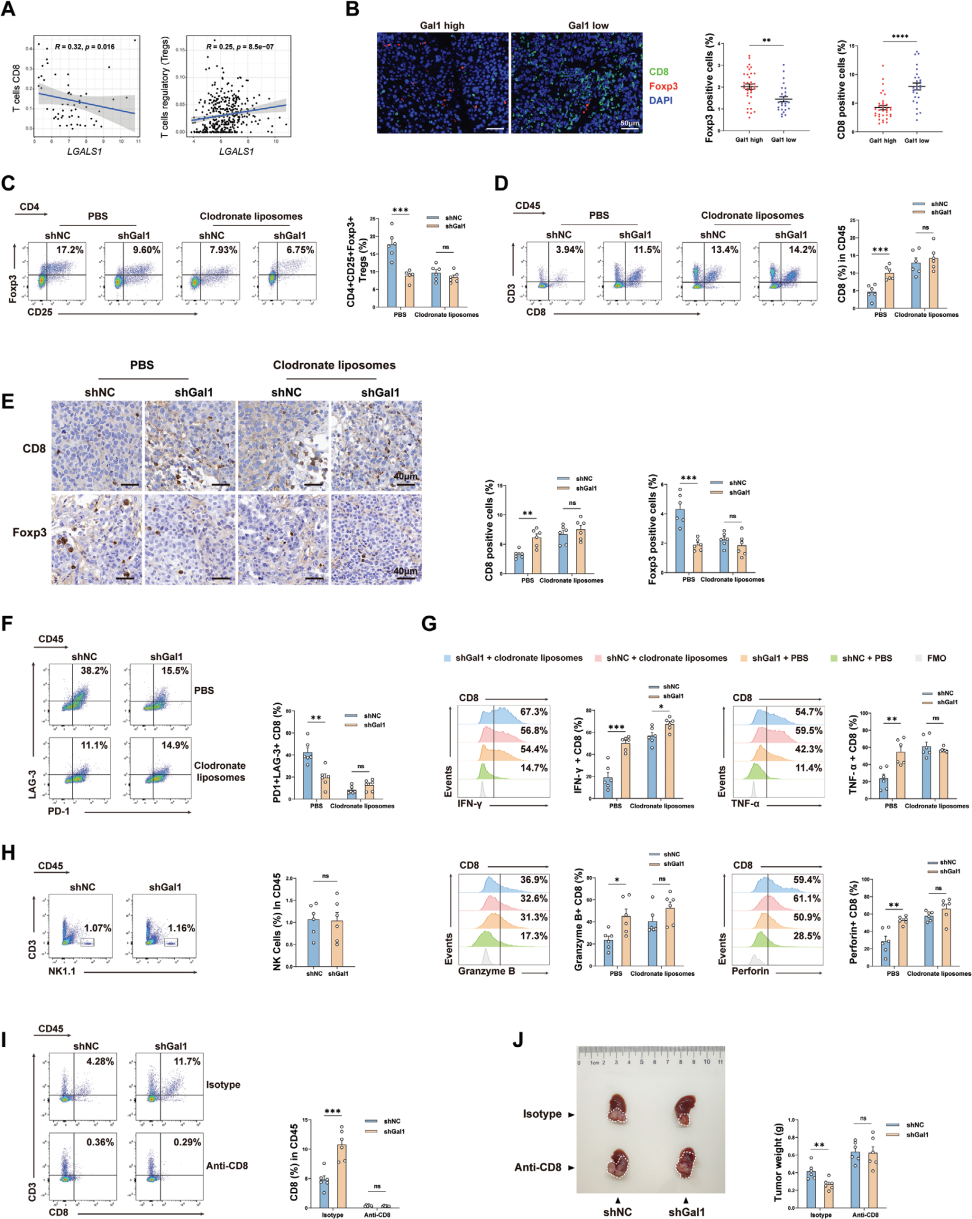
TAMs are critical mediators in the establishment of immunosuppressive TME induced by Gal1. A) Analysis of the correlation between Gal1 expression and CD8^+^ T cell infiltration (left) and Treg infiltration (right) in TCGA database. B) Representative images (upper) of IF staining with Foxp3 (red), CD8 (green), DAPI (blue) in HCC tissues with high Gal1 expression (IHC staining score ≥ 6) and low Gal1 expression (IHC staining score < 6); (lower) quantification of Foxp3^+^ and CD8^+^ cells in HCC tissues. C) Flow cytometry analysis of CD4^+^CD25^+^Foxp3^+^ Treg infiltration in orthotopic HCC mouse models with or without macrophage depletion (n = 6). D) Flow cytometry analysis of the proportion of CD8^+^ T cells in orthotopic HCC mouse models (n = 6). E) Representative IHC staining images and quantification of Foxp3^+^ and CD8^+^ cells in mouse tumor tissues (n = 6). F) Flow cytometry analysis of the proportion of PD‐1^+^LAG‐3^+^CD8^+^ T cells in orthotopic HCC mouse models (n = 6). G) Percentage of IFN‐γ^+^, TNF‐α^+^, granzyme B^+^ and perforin^+^ CD8^+^ T cells assessed by flow cytometry (n = 6). H) Flow cytometry analysis of NK1.1^+^ cell infiltration in the orthotopic HCC mouse models (n = 6). I) The proportion of CD8^+^ T cells in the orthotopic HCC mouse models treated with isotype or anti‐CD8 mAb (n = 6). J) Representative image of tumor (left) and tumor weight (right) in the orthotopic HCC mouse models treated with isotype or anti‐CD8 mAb (n = 6). Data were presented as mean ± SEM. ^*^ *P* < 0.05; ^**^ *P* < 0.01; ^***^ *P* < 0.001; ^****^ *P* < 0.0001.

To further evaluate the impact of Gal1 on the tumor immune microenvironment, we analyzed immune infiltration in HCC samples from the TCGA database. We observed a negative correlation between tumor Gal1 expression and CD8^+^ T cells, while the Gal1 expression was positively correlated with Tregs within the TME, suggesting that Gal1 contributed to developing an immunosuppressive environment in HCC (**Figure** **2**A). Consistent with this, IHC and immunofluorescence (IF) assays on tumor tissue samples obtained from HCC patients also demonstrated reduced CD8^+^ T cell infiltration and increased Foxp3^+^ Treg infiltration in HCC samples with high Gal1 expression compared to those with low Gal1 expression (Figure 2B). These were further corroborated through flow cytometry and IHC staining analyses of orthotopic mice tumor samples (shGal1 or shNC) (Figure 2C–E). Additionally, CD8^+^CD122^+^PD‐1^+^ Tregs are emerging as an important subset of T suppressors, and our results revealed that there is no significant difference between shGal1 and shNC liver orthotopic HCC mouse models (Figure S3, Supporting Information). Moreover, we found that Gal1 knockdown alleviated CD8^+^ T cell exhaustion and enhanced CD8^+^ T cell function, indicated by pronounced expression of tumor necrosis factor‐α (TNF‐α), interferon‐γ (IFN‐γ), perforin and granzyme B (Figure 2F,G). Notably, the infiltration of NK cells, which possess certain tumor‐killing capabilities, remained unaffected following Gal1 knockdown (Figure 2H). However, the tumor inhibition effect elicited by Gal1 knockdown was nullified when CD8^+^ T cells were depleted using an anti‐CD8 monoclonal antibody, indicating that Gal1 primarily acts by suppressing CD8^+^ T cells (Figure 2I,J). Furthermore, the impact of Gal1 knockdown on Treg infiltration, as well as tumor‐infiltrating CD8^+^ T cell quantity, exhaustion, and functionality, was rescued when macrophages were depleted with clodronate liposomes (Figure 2C–G). Consequently, we propose that Gal1‐induced TAMs ultimately give rise to an increase in Tregs and suppression in the infiltration and functionality of CD8^+^ T cells. CD4^+^CD25^+^Foxp3^+^ Tregs have a strong potential to hinder the accumulation and effector function of tumor‐infiltrating CD8^+^ T cells through a variety of mechanisms.^[^ ^28^ ^]^ On the other hand, TAMs could recruit CD4^+^CD25^+^Foxp3^+^ Tregs into the TME by releasing multiple chemokines, including CCL20, CCL22, to facilitate the establishment of tumor immunosuppressive micro‐environment.^[^ ^11^, ^29^ ^]^ Therefore, we supposed that TAMs might induce the suppressed accumulation and function of CD8^+^ T cells through recruiting CD4^+^CD25^+^Foxp3^+^ Tregs into the TME of HCC patients with high Gal1 expression.

### 2.3 Tumor‐Derived Gal1 Facilitates Tregs Recruitment through Upregulating CCL20 Expression in TAMs

**Figure 3:**
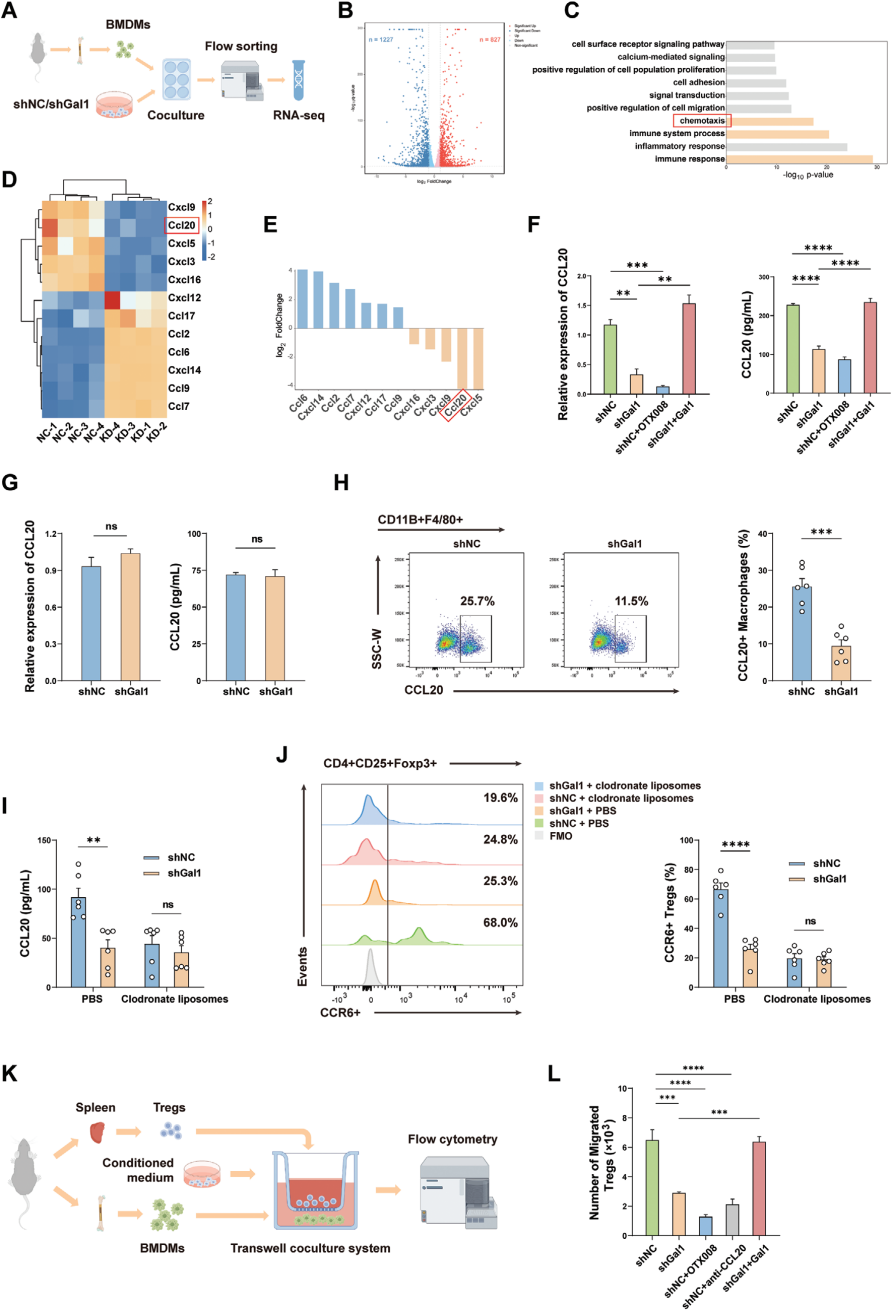
Tumor‐derived Gal1 facilitates Tregs recruitment through upregulating CCL20 expression in TAMs. A) Schematic workflow showing BMDM and tumor cell co‐culture and RNA‐sequencing. B) Volcano plot of differentially expressed genes based on RNA‐sequencing. C) Pathway enrichment analysis of RNA‐sequencing dataset. D) Cluster heatmap of differentially expressed chemokines based on RNA‐sequencing. E) Changes in chemokine levels according to RNA‐sequencing. F) qPCR analysis of CCL20 expression in BMDMs cultured with conditioned medium from shGal1 or shNC hepa1‐6 cells for 24 hours (left, n = 3); ELISA analysis of CCL20 protein expression in supernatants (right, n = 3). G) qPCR analysis of CCL20 expression in shGal1 or shNC hepa1‐6 cells (left, n = 3); ELISA analysis of CCL20 protein expression in the supernatants from shGal1 or shNC hepa1‐6 cells (right, n = 3). H) Flow cytometry analysis of CCL20 expression in TAMs in the orthotopic HCC mouse models (n = 6). I) The proportion of CCR6^+^ Tregs in the orthotopic HCC mouse models assessed by flow cytometry (n = 6). J) ELISA analysis of CCL20 protein level in tumor tissues from the orthotopic HCC mouse models with or without macrophage depletion (n = 6). K) Schematic diagram of Treg chemotaxis assay. L) Flow cytometry analysis of the number of migrated CD4^+^CD25^+^Foxp3^+^ Tregs (n = 3). Data were presented as mean ± SEM. ^*^ *P* < 0.05; ^**^ *P* < 0.01; ^***^ *P* < 0.001; ^****^ *P* < 0.0001.

To investigate the mechanism by which tumor‐derived Gal1 enhances the immunosuppressive capacity of macrophages, bone marrow‐derived macrophages (BMDMs) were incubated with shGal1 or shNC hepa1‐6 cells. After 24 hours, macrophages were isolated using flow sorting for transcriptome sequencing (**Figure** **3**A; Figure S2B, Supporting Information). Compared to the control group, BMDMs co‐incubated with shGal1 exhibited a down‐regulation of 1227 genes and an up‐regulation of 827 genes (Figure 3B). Notably, the most significantly altered pathways were enriched in immune response and chemotaxis (Figure 3C). The most significantly decreased chemokines were CCL20 and CXCL5 with Gal1 knockdown (Figure 3D,E). Given the known association between CCL20 and Treg chemotaxis,^[^ ^29^, ^30^ ^]^ we postulated that Gal1 might enhance the secretion of CCL20 by macrophages to recruit Tregs. Consistent with the transcriptome trend, qPCR and ELISA analysis of BMDMs incubated in conditioned medium (CM) from the shGal1 group showed decreased CCL20 level compared to the shNC group (Figure 3F). Simultaneously, recombinant Gal1 was introduced into the CM of shGal1, resulting in elevated expression and secretion of CCL20, while the addition of Gal1 inhibitor OTX008 to the shNC CM showed a converse effect (Figure 3F). Additionally, the expression of CCL20 in tumor cells was measured between shGal1 and shNC groups, revealing no significant difference (Figure 3G). This result suggested that the observed alteration in CCL20 secretion level in this system is primarily attributed to macrophages. Following this, flow cytometry analysis revealed a notable reduction in the secretion of CCL20 by TAMs within the TME of orthotopic tumors originating from inoculation of shGal1 cells, as compared to the control group (Figure 3H). Consistent with this, the CCL20 level in orthotopic tumor tissues measured by ELISA demonstrated similar trends (Figure 3I). However, the depletion of macrophages in vivo resulted in similar levels of CCL20 expression between shGal1 and shNC tumors (Figure 3H,I). In conclusion, our findings indicated that tumor‐derived Gal1 played a significant role in promoting the secretion of CCL20 by TAMs, and these changes of CCL20 were consistent with the changes of CD4^+^CD25^+^Foxp3^+^ Treg infiltration in TME aforementioned.

In order to exclude the impact of Gal1 on the proliferation of Tregs, we incubate splenic CD4^+^CD25^+^Foxp3^+^ Tregs in the CM from shGal1 and shNC. The resulting proliferation levels were found to be comparable (Figure S2C,D, Supporting Information). It is worth noting that CCR6 is presently recognized as the sole receptor for CCL20.^[^ ^31^ ^]^ And the flow cytometry analysis revealed that Gal1 knockdown significantly decreased the proportion of CCR6^+^CD4^+^CD25^+^Foxp3^+^ Tregs within the TME. Interestingly, this effect was mitigated when macrophages were previously subjected to depletion (Figure 3J). Furthermore, we conducted a Treg chemotaxis assay in vitro and demonstrated that BMDMs cultured in the CM from shNC exhibited stronger ability to attract Tregs than shGal1, and this effect was blunted through the application of anti‐CCL20 neutralizing antibody and OTX008 (Figure 3K,L). Hence, we propose that the increased Treg infiltration in the TME primarily occurs due to the enhanced secretion of CCL20 by Gal1‐induced TAMs.

### 2.4 Tregs Recruited by Gal1‐Induced TAMs Contribute to Impaired Functionality of CD8^+^ T Cells

**Figure 4:**
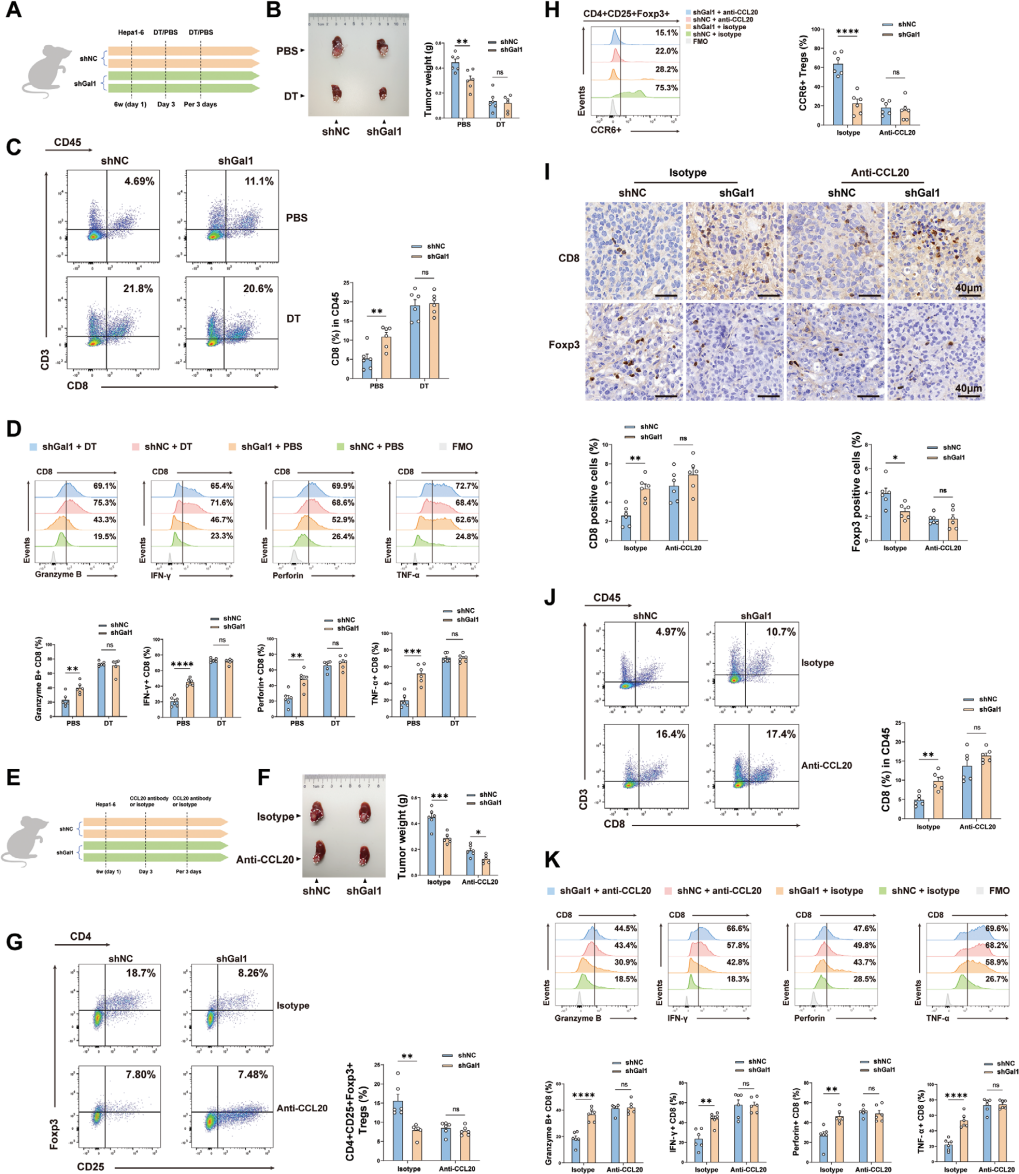
Tregs recruited by Gal1‐induced TAMs contribute to impaired functionality of CD8^+^ T cells. A) Flow chart of Treg depletion in *Foxp3DTR* mice. B) Representative image of tumor (left) and tumor weight (right) in the orthotopic HCC mouse models with or without Treg depletion (n = 6). C) Infiltration of CD8^+^ T cells in the orthotopic HCC mouse models was determined by flow cytometry (n = 6). D) Flow cytometry analysis of IFN‐γ^+^, TNF‐α^+^, granzyme B^+^ and perforin^+^ CD8^+^ T cells in the orthotopic HCC models (n = 6). E) Schematic flow diagram of shNC and shGal1 cell implantation and CCL20 neutralization. F) Representative image of tumor (left) and tumor weight (right) in the orthotopic HCC models treated with isotype or anti‐CCL20 antibody (n = 6). G) Flow cytometry analysis of CD4^+^CD25^+^Foxp3^+^ Tregs in the orthotopic HCC models (n = 6). H) The proportion of CCR6^+^ Tregs in the orthotopic HCC models was assessed by flow cytometry (n = 6). I) Representative IHC staining images (upper) and quantification (lower) of Foxp3^+^ and CD8^+^ cells in mouse tumor tissues (n = 6). J) Flow cytometry analysis of the infiltration of CD8^+^ T cells in the orthotopic HCC mouse models (n = 6). K) Flow cytometry analysis of the expression of anti‐tumor cytokines (IFN‐γ, TNF‐α, granzyme B and perforin) in CD8^+^ T cells in the orthotopic HCC mouse models (n = 6). Data were presented as mean ± SEM. ^*^ *P* < 0.05; ^**^ *P* < 0.01; ^***^ *P* < 0.001; ^****^ *P* < 0.0001.

To examine whether the Gal1‐induced suppression of CD8^+^ T cell‐mediated antitumor response in the TME is linked to CD4^+^CD25^+^Foxp3^+^ Tregs, Treg‐depleted mice was established by administering diphtheria toxin (DT) intraperitoneally to *Foxp3DTR* mice (Figure S2E, Supporting Information). Hepa1‐6 cells (shGal1 or shNC) were orthotopically inoculated into the livers of Treg‐depleted and control mice, and subsequent measurements were taken to assess tumor weight, CD8^+^ T cell infiltration and functionality (**Figure** **4**A). Whereas depletion of CD4^+^CD25^+^Foxp3^+^ Tregs inhibited tumor growth and enhanced infiltration and function of CD8^+^ T cells in vivo, Gal1 knockdown failed to further improve the anti‐tumor effect in Treg‐depleted mice (Figure 4B–D, Figure S2F, Supporting Information). These results implied that CD4^+^CD25^+^Foxp3^+^ Tregs played a major role in Gal1‐induced pro‐tumoral effects. Furthermore, we injected an anti‐CCL20 neutralizing antibody into the tumor‐bearing mice by tail vein to block the CCL20‐CCR6 axis. We found that the proportion of tumor‐infiltrating CD4^+^CD25^+^Foxp3^+^ Tregs, especially the CCR6^+^ Treg subpopulation, was attenuated (Figure 4E,G–I). This intervention allowed us to identify CD4^+^CD25^+^Foxp3^+^CCR6^+^ Tregs as the primary source of Gal1‐induced Treg infiltration. Additionally, the tumor weight, infiltration level, and anti‐tumor activity of CD8^+^ T cells in the shNC tumors treated with anti‐CCL20 antibody were comparable to those in the shGal1 tumors treated with anti‐CCL20 antibody (Figure 4F,I–K). In contrast, administering CCL20 via intratumoral injection in subcutaneous HCC mouse models restored tumor growth, reduced the CD8^+^ T cell infiltration, increased the frequency of CD4^+^CD25^+^Foxp3^+^ Tregs and CCR6^+^ Tregs in shGal1 tumors (Figure S4, Supporting Information). These results implied that the recruited CD4^+^CD25^+^Foxp3^+^CCR6^+^ Tregs were accountable for the diminished anti‐tumor efficacy of CD8^+^ T cells and the subsequent progression of the tumor. In conclusion, our findings suggested that tumor‐derived Gal1 promoted the secretion of CCL20 by TAMs, thereby recruiting Tregs as collaborators in tumor development and impeding the function and infiltration of CD8^+^ T cells.

### 2.5 Gal1 Upregulates CCL20 through Activation of the PI3K/AKT/NF‐κB Signaling Pathway in TAMs

**Figure 5:**
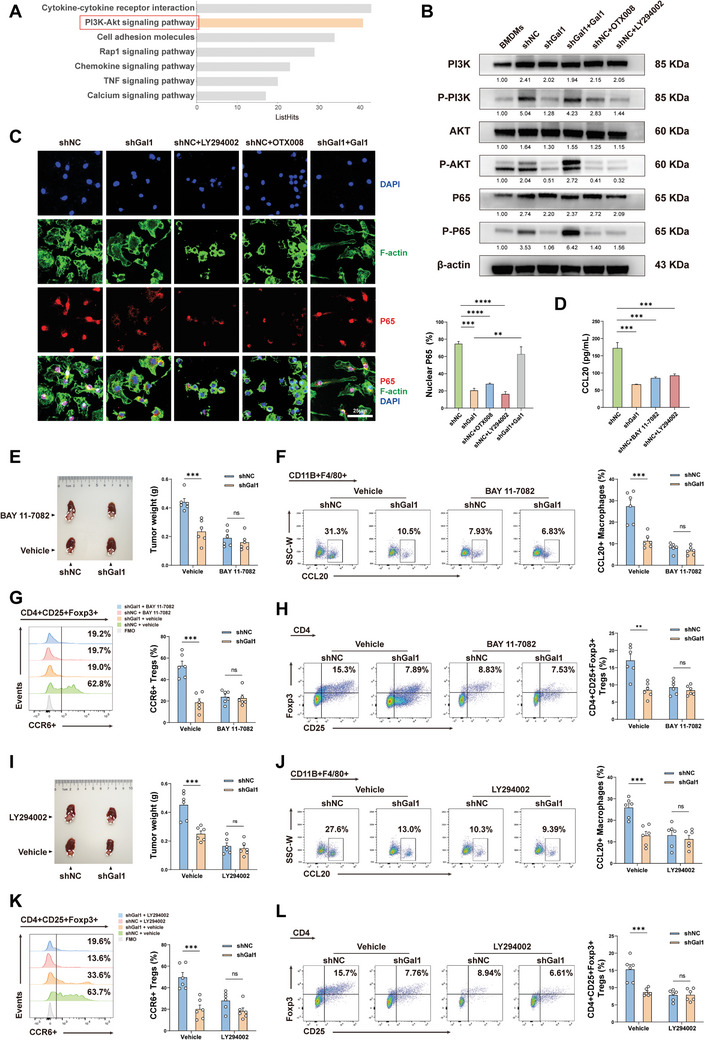
Gal1 upregulates CCL20 through activation of the PI3K/AKT/NF‐κB signaling pathway in TAMs. A) Pathway enrichment analysis of differentially expressed genes based on RNA‐sequencing. B) Protein levels of PI3K/AKT/NF‐κB pathway in BMDMs incubated in the conditioned medium for 24 hours were determined by western blot. C) Representative IF images (left) and quantification (right) of P65 nuclear translocation in BMDMs incubated in the conditioned medium (n = 3). D) ELISA analysis of CCL20 protein expression in supernatants of BMDMs cultured with conditioned medium from shGal1 or shNC hepa1‐6 cells for 24 hours (n = 3). E) Representative image of tumor (left) and tumor weight (right) in the orthotopic HCC models treated with vehicle or BAY 11–7082 (n = 6). F) CCL20 expression in TAMs in the orthotopic HCC models was measured by flow cytometry (n = 6). G) Flow cytometry analysis of the proportion of CCR6^+^ Tregs in the orthotopic HCC models (n = 6). H) Flow cytometry analysis of CD4^+^CD25^+^Foxp3^+^ Tregs in the orthotopic HCC models (n = 6). I) Representative image of tumor (left) and tumor weight (right) in the orthotopic HCC models treated with vehicle or LY294002 (n = 6). J) Flow cytometry analysis of CCL20 expression in TAMs in the orthotopic HCC models (n = 6). K) Flow cytometry analysis of the proportion of CCR6^+^ Tregs in the orthotopic HCC models (n = 6). L) Flow cytometry analysis of CD4^+^CD25^+^Foxp3^+^ Tregs in the orthotopic HCC models (n = 6). Data were presented as mean ± SEM. ^*^ *P* < 0.05; ^**^ *P* < 0.01; ^***^ *P* < 0.001; ^****^ *P* < 0.0001.

Previous research demonstrated that NF‐κB served as the specific transcription factor binding to the CCL20 gene promoter and was responsible for facilitating CCL20 expression.^[^ ^32^ ^]^ Concurrently, pathway enrichment analysis of the transcriptome results revealed a significant concentration of differentially expressed genes within the PI3K‐AKT pathway (**Figure** **5**A). Consequently, we propose that Gal1 may enhance CCL20 expression by activating the PI3K/AKT/NF‐κB pathway in TAMs. Following this, we incubated BMDMs in the CM from shGal1 or shNC cells, respectively, for 24 hours, and the results demonstrated that knockdown of Gal1 in tumor cells contributed to the inhibition of PI3K, AKT, and P65 phosphorylation in macrophages, as well as the hindrance of P65 nuclear translocation (Figure 5B,C). However, adding recombinant Gal1 protein to the CM from shGal1 cells reversed this phenomenon. Conversely, the addition of the Gal1 inhibitor OTX008 to the CM of the shNC inhibited the activation of this pathway (Figure 5B,C). These results suggested that tumor‐derived Gal1 was a key regulator of this process. Additionally, the introduction of the PI3K inhibitor LY294002 to the CM from shNC resulted in a significant reduction in the phosphorylation levels of PI3K and AKT, as well as the inhibition of nuclear translocation and phosphorylation of P65 (Figure 5B,C). Based on these findings, we proposed that Gal1 could activate NF‐κB in macrophages through the PI3K‐AKT pathway. Furthermore, in vitro ELISA assays showed that the addition of the NF‐κB inhibitor BAY 11–7082 and the PI3K inhibitor reduced the CCL20 content in the culture supernatant (Figure 5D). Conversely, ELISA assays and flow cytometry analysis showed that adding NF‐κB activator 1 and PI3K activator 740 Y‐P restored CCL20 expression in vitro (Figure S5, Supporting Information). These results suggested that the activation of the PI3K‐AKT‐NF‐κB pathway could facilitate the secretion of CCL20. To further validate this conclusion, orthotopic HCC murine models injected with shGal1 or shNC were administered NF‐κB and PI3K inhibitors (Figure 5E,I). The results demonstrated that inhibiting the activation of this pathway effectively diminished CCL20 secretion in TAMs and hindered the recruitment of CCR6^+^ Tregs, consequently reducing the proportion of Tregs and impeding tumor growth (Figure 5E–L). Moreover, the inhibition of this pathway blunted the diminished CCL20 secretion and Treg recruitment induced by Gal1 knockdown (Figure 5E–L). Collectively, the activation of the PI3K‐AKT‐NF‐κB pathway in TAMs induced by tumor‐derived Gal1 was a significant contributor to the secretion of CCL20 and the recruitment of Tregs in HCC.

### 2.6 Blocking Gal1 Reverses the Anti‐PD1 Resistance in HCC

**Figure 6:**
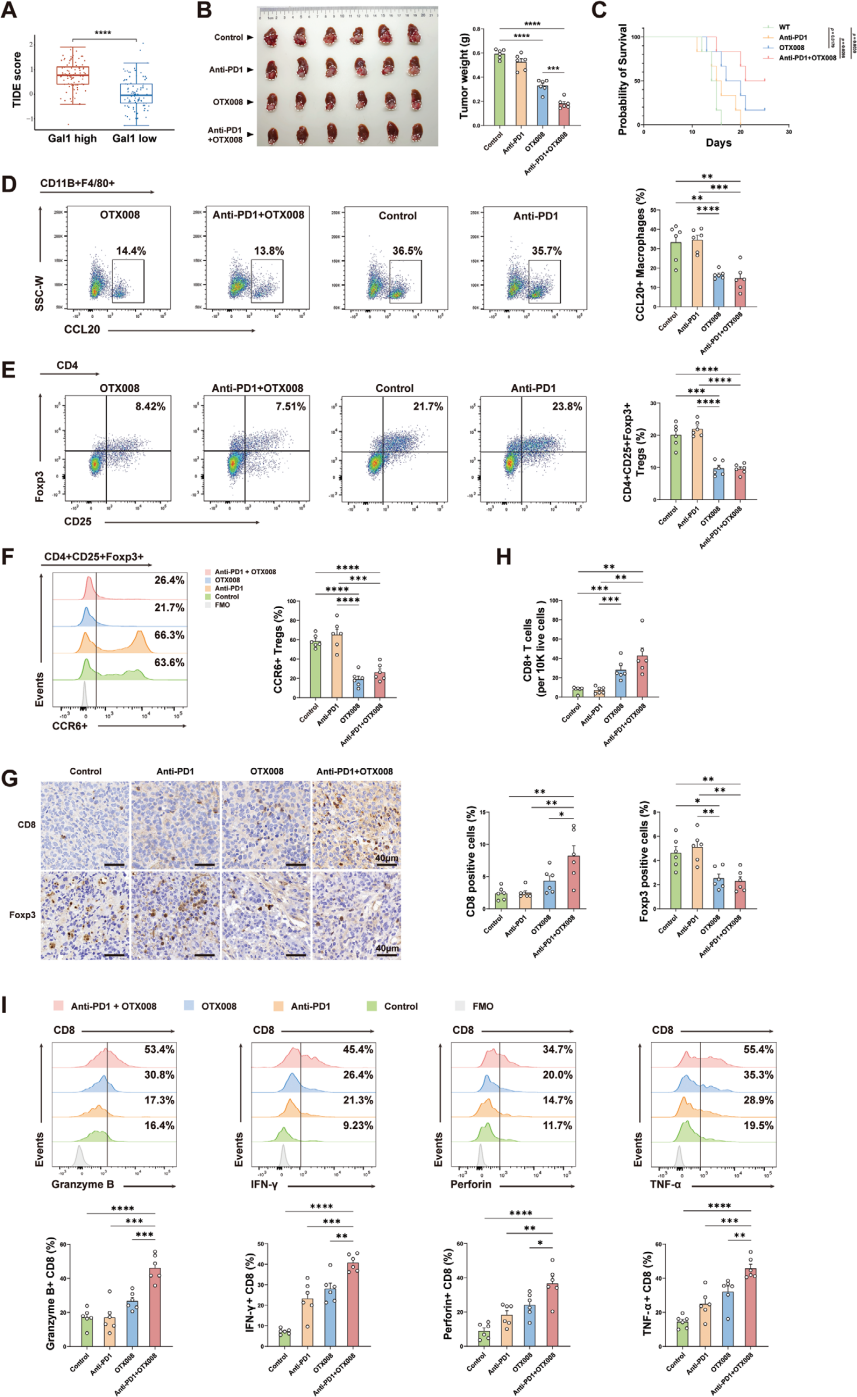
Blocking Gal1 reverses the anti‐PD1 resistance in HCC. A) Comparison of TIDE scores between Gal1 high expression and Gal1 low expression groups in TCGA cohort. B) Image of HCC tumors (left) and quantification of tumor weight (right) in orthotopic HCC models treated with OTX008, anti‐PD‐1 mAb or combination therapy (n = 6). C) Overall survival of tumor‐bearing mice treated with monotherapy or combination therapy (n = 6). D) Flow cytometry analysis of CCL20 expression in TAMs in the orthotopic HCC models with different treatment (n = 6). E) Flow cytometry analysis of CD4^+^CD25^+^Foxp3^+^ Tregs in the orthotopic HCC models (n = 6). F) The percentage of CCR6^+^ Tregs in the orthotopic HCC models was determined by flow cytometry (n = 6). G) Representative IHC staining images (upper) and quantification (lower) of Foxp3^+^ and CD8^+^ cells in the orthotopic HCC models with different treatment (n = 6). H) The number of live CD8^+^ T cells in the orthotopic HCC models was assessed by flow cytometry (n = 6). I) Intracellular staining of granzyme B, perforin, IFN‐γ and TNF‐α in CD8^+^ T cells in the orthotopic HCC models was determined by flow cytometry (n = 6). Data were presented as mean ± SEM. ^*^ *P* < 0.05; ^**^ *P* < 0.01; ^***^ *P* < 0.001; ^****^ *P* < 0.0001.

Numerous studies demonstrated that Tregs within the TME could impede the functionality of CD8^+^ T cells either through direct or indirect mechanisms, ultimately resulting in the ineffectiveness of anti‐PD‐1 therapy.^[^ ^28^ ^]^ In addition, our results revealed that Gal1‐induced TAMs were capable of attracting a considerable quantity of Tregs into the TME. Moreover, the TIDE algorithm was applied to predict the potential ICB response among HCC patients with varying levels of Gal1 expression.^[^ ^33^ ^]^ The results suggested that those with low Gal1 expression might exhibit a more favorable response to anti‐PD‐1 therapy (**Figure** **6**A). Given the prevalence of elevated Gal1 expression in liver cancer patients, it was postulated that targeting Gal1 could potentially improve the efficacy of anti‐PD‐1 therapy in the HCC treatment. To prove our hypothesis, orthotopic mouse models of HCC were established and subjected to treatment with Gal1 inhibitor OTX008, anti‐PD‐1 mAb, or a combination of both. In comparison to the control group, anti‐PD‐1 therapy yielded suboptimal results, whereas Gal1 blockade demonstrated superior efficacy. Notably, the combination of anti‐PD‐1 mAb and Gal1 inhibitor exhibited a pronounced inhibition of tumor growth and a notable enhancement in mouse survival (Figure 6B,C). Furthermore, flow cytometry analysis demonstrated that the combination therapy effectively suppressed the secretion of CCL20 by TAMs, which subsequently decreased the recruitment of CCR6^+^ Tregs and the proportion of Tregs in the TME (Figure 6D–G). Additionally, there was an observed increase in the infiltration of CD8^+^ T cells, accompanied by an enhancement of their cytotoxic capabilities (Figure 6G–I). Consequently, blocking the Gal1‐mediated interaction between the tumor cells and TAMs further improved the efficacy of anti‐PD‐1 therapy, and Gal1 emerged as an attractive novel target for combination therapy of HCC patients.

## 3 Discussion

Macrophages are the predominant infiltrating innate immune cells within the immune microenvironment of liver cancer.^[^ ^14^ ^]^ Contrary to previous beliefs regarding their involvement in anti‐tumor immunity, TAMs within the TME often promote tumor development, as tumor cells are capable of converting these innate immune cells into a pro‐tumor phenotype through various mechanisms. Prior research indicated that tumor cells had the ability to attract macrophages by releasing various cytokines, including CSF‐1, VEGFA, and CCL2.^[^ ^11^, ^34^ ^]^ Meanwhile, tumor cells and other cells within the TME could induce a pro‐tumor phenotype in TAMs through the secretion of cytokines such as IL‐4, IL10, and CSF‐1.^[^ ^11^, ^35^, ^36^ ^]^ However, the complexity of the tumor microenvironment and heterogeneity of TAMs contributed to intricate regulatory interactions between tumor cells and macrophages, necessitating further exploration of specific mechanisms. Here, we demonstrated that tumor‐derived Gal1 could enhance the tumor‐promoting capability of TAMs in HCC.

Gal1, an immunosuppressive protein secreted by tumor cells, was implicated in the advancement of various types of cancer, such as breast cancer, melanoma, head and neck cancer, and glioma.^[^ ^22^, ^37^, ^38^, ^39^ ^]^ It was previously demonstrated that Gal1 was able to facilitate the infiltration of Tregs within the TME, although the precise underlying mechanism remained to be elucidated.^[^ ^37^, ^40^ ^]^ Consist with this, we further revealed an association between tumor‐secreted Gal1 and Tregs through analysis of tumor samples obtained from patients with HCC and orthotopic murine models. Interestingly, following the depletion of macrophages in mice, the relationship between Gal1 and Treg infiltration as well as tumor development was disrupted, suggesting that Gal1 could exert an immunosuppressive effect through its interaction with macrophages. Analysis of transcriptome data from BMDMs incubated with shNC or shGal1 hepa1‐6 cells revealed that tumor‐derived Gal1 was capable of stimulating CCL20 secretion by macrophages. This finding aligned with previous research by Samaniego et al., who observed that tumor‐derived factors present in the tissue supernatant of primary melanoma were able to promote CCL20 secretion by macrophages.^[^ ^41^ ^]^ However, the specific factors responsible for this effect were not identified. Through enrichment analysis and a comprehensive set of in vitro and in vivo experiments, we confirmed that Gal1 stimulated the secretion of CCL20 by activating the PI3K/AKT/NF‐κB pathway in TAMs. In reference to CCL20, CCR6 is the only receptor that has been identified.^[^ ^31^ ^]^ In addition, it was previously shown that a majority of Tregs present in the TME of HCC were CCR6 positive,^[^ ^42^ ^]^ which was consistent with our findings. Depletion of macrophages in mice or blockade of CCL20 by a neutralizing antibody led to a reduction in CCR6^+^ Treg recruitment and subsequently in the proportion of CD4^+^CD25^+^Foxp3^+^ Tregs. Therefore, we proposed that Gal1 facilitated Treg infiltration primarily through stimulating CCL20 secretion by TAMs.

Tumor‐infiltrating Tregs have the potential to hinder effector T cell function through various mechanisms, including the release of suppressive cytokines like IL‐10, IL‐35, and TGF‐β, as well as the expression of immunoinhibitory surface molecules such as CD39, CD73, and CTLA‐4.^[^ ^28^, ^43^ ^]^ This characteristic may elucidate the mechanism behind the decreased infiltration of CD8^+^ T cells and their dysfunction resulting from Gal1. Furthermore, upon systemic depletion of Tregs in *Foxp3DTR* mice or inhibition of CCR6^+^ Treg recruitment with an anti‐CCL20 neutralizing antibody, the CD8^+^ T cell cytotoxicity increase following Gal1 knockdown was negated. These findings suggested that Tregs recruited by Gal1‐induced TAMs might be the main subset exerting the suppressive effect against CD8^+^ T cells.

Currently, the efficacy of PD1 blockade in patients with HCC is limited, with only a minority experiencing benefit from it. Therefore, there is a pressing clinical imperative to enhance the effectiveness of immunotherapy and identify predictive biomarkers. In our investigation, blockade of Gal1, which contributed to potent immunosuppressive effect through interaction with TAMs in the liver cancer TME, resulted in a reduction in Treg infiltration and an enhancement of CD8^+^ T cell infiltration and function, thus leading to a notable improvement in the effectiveness of PD1 therapy combined with Gal1 inhibitor OTX008. Consequently, targeting the interaction between tumor cells and TAMs via Gal1 is expected to improve the response and efficacy of immune checkpoint blockade (ICB) treatment in HCC patients.

**Figure 7:**
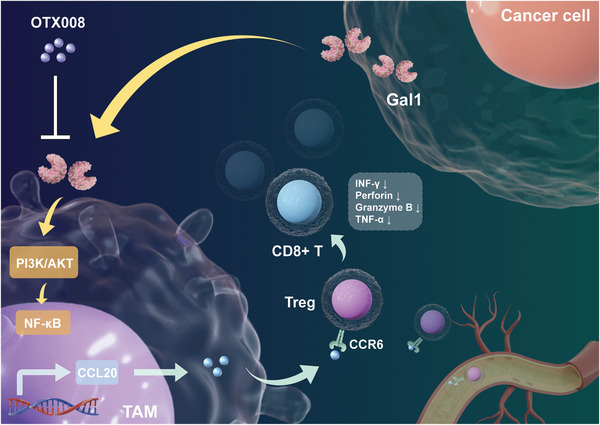
Schematic representations of Gal1‐induced TAMs in maintaining the immunosuppressive tumor microenvironment in HCC.

In summary, a newly identified interaction mediated by tumor‐derived Gal1 between tumor cells and TAMs has been recognized. This interaction facilitates the recruitment of CCR6^+^ Tregs by macrophages, subsequently leading to the suppression of the anti‐tumor activity of CD8^+^ T cells. Additionally, targeting Gal1 has been shown to enhance the efficacy of anti‐PD1 therapy, suggesting a novel combination treatment strategy and predictive biomarker for immunotherapy in HCC (**Figure** **7**).

## 4 Experimental Section

### Human Samples

A total of 62 cancer tissues and 38 para‐cancerous tissues were harvested from patients with HCC who underwent curative resection in the First Affiliated Hospital, Zhejiang University School of Medicine. These samples were used for survival analysis, immunohistochemistry, and immunofluorescence. This study was approved by the Ethics Committee of the First Affiliated Hospital, Zhejiang University School of Medicine (IIT20240142A). Clinical information was summarized in Table S1 (Supporting Information).

### Cell Culture and Transfection

The detailed methods were provided in the supporting information.

### Mouse Tumor Models

Mouse tumor model establishment was described in detail in the supporting information. Experimental mice were housed in SPF conditions. All animal experiments were approved by the animal Ethics Committee of the First Affiliated Hospital, Zhejiang University School of Medicine (2024‐34) and followed the National Research Council's Guide for the Care and Use of Laboratory Animals.

### Mouse Treatment

For depletion of macrophages, 1 day before tumor inoculation, mice were injected via the tail vein with 200 µL mouse^−1^ clodronate liposomes or control PBS liposomes (40337ES10 or 40338ES10, Yeasen Biotechnology) every 4 days. For depletion of CD8^+^ T cells, mice were injected intraperitoneally with anti‐mouse CD8α antibody (12.5 mg kg^−1^, BE0061, BioXcell) or isotype antibody (BE0090, BioXcell) every 3 days after tumor inoculation. For depletion of Tregs, 2 days after tumor implantation, C57BL/6J *Foxp3DTR* mice were performed by intraperitoneal injection of diphtheria toxin (12.5 µg kg^−1^, 15047A1, List Labs) or PBS every 3 days. For neutralization of CCL20 in vivo, tumor‐bearing mice were injected intraperitoneally with 10µg dose^−1^ anti‐mouse CCL20 antibody (AF760, R&D Systems) or isotype every 3 days from the third day. For combination treatment, PD‐1 blockade in tumor‐bearing mice was performed from day 3 by intraperitoneal injection of anti‐PD‐1 (100µg dose^−1^, BE0146, BioXcell) or isotype every 3 days; Gal1 inhibition in mice was performed from day 2 by intraperitoneal injection of OTX008 (5 mg/kg, HY‐19756, MCE) or vehicle every 3 days. For PI3K inhibition in vivo, mice received intraperitoneal injection of LY294002 (HY‐10108, MCE) every 3 days, starting from the third day after tumor implantation. For NF‐κB inhibition in vivo, mice received intraperitoneal injection of BAY 11–7082 (HY‐13453, MCE) every 3 days, starting from the third day after tumor implantation.

### Tumor Processing and Flow Cytometry

Tumor tissues were digested and processed into single‐cell suspensions using the Tumor Dissociation Kit (Miltenyi Biotec, 130‐096‐730) according to the manufacturer's guidelines. CD8^+^ T cells were enriched by anti‐CD8 magnetic beads (551516, BD Biosciences) and stimulated by Leukocyte Activation Cocktail (550583, BD Biosciences) for 4–6 hours at 37°C prior to staining. CD4^+^CD25^+^Foxp3^+^ Tregs were enriched by anti‐CD4 magnetic beads (551539, BD Biosciences) prior to staining. Cells were all incubated with TruStain FcX™ (Biolegend, 101319) for 15 min at 4°C to block Fc receptors prior to cell surface staining. For intracellular cytokine staining, cells were fixed and permeabilized using BD Cytofix/Cytoperm Kit (BD Biosciences, 554715). Intracellular staining for Foxp3 was performed after fixation and permeabilization using the Foxp3 transcription factor staining kit (eBioscience, 00‐5523‐00). The precise details of antibodies and staining were given in the supporting information.

### Immunohistochemistry and Immunofluorescence

Staining was performed on 3‐µm slides, and five fields were randomly chosen to evaluate staining. The criteria for IHC scoring were shown in supplementary Figure S1 (Supporting Information). Detailed procedures were provided in the supporting information.

### Western Blot

The detailed procedures of western blot were in the supporting information.

### BMDMs Generation

Bone marrow‐derived macrophages (BMDMs) were harvested from the thig‐bones of C57BL/6J mice and cultured in the RPMI‐1640 medium containing 10% heat‐inactivated FBS, 1% penicillin‐streptomycin and 25 ng/ml M‐CSF (315‐02, PeproTech). Cells were cultured for 6 days with the medium changed every 48 h for further use.

### Tregs Isolation

Tregs were isolated from single‐cell suspensions of mouse spleens using the mouse CD25 regulatory T cell positive selection kit (18782, STEMCELL) following the manufacturer's instructions. These cells were then cultured in the RPMI‐1640 medium containing 10% heat‐inactivated FBS, 1% penicillin‐streptomycin, 2000U ml^−1^ IL‐2 (212‐12, PeproTech), 50 µM β‐mercaptoethanol, 25 mM HEPES and 1% sodium pyruvate for further use.

### RNA‐Sequence and Analysis

The detailed procedures were provided in the supporting information.

### RNA Extraction and Quantitative RT‐PCR

The procedures of RNA extraction and quantitative RT‐PCR were described in the supporting information. The primers used were in Table S2 (Supporting Information).

### ELISA

The CCL20 concentration in tumor tissues and the supernatant of different groups was measured using the mouse CCL20 ELISA kit (ELM‐MIP3a, Raybiotech) in accordance with the manufacturer's instructions.

### Treg Migration Assay

Transwell chambers with 5 µm polycarbonate filters (725201, NEST) were used in the Treg migration assay. 1 × 10^5^ isolated CD4^+^CD25^+^Foxp3^+^ Tregs were added to the upper chamber, 2 × 10^5^ BMDMs were seeded in the lower chamber wherein CM from shNC or shGal1 cells was also added. Furthermore, OTX008 (50 µg ml^−1^), anti‐mouse CCL20 antibody (20 µg ml^−1^, ab9829, Abcam) or recombinant Gal1 (10 µg ml^−1^, 450‐39, PeproTech) was added to these cells. After 48 h of incubation at 37 °C, living CD4^+^CD25^+^Foxp3^+^ Tregs that had migrated through the filter were measured by flow cytometry.

### CFSE Expansion Assay

The isolated CD4^+^CD25^+^Foxp3^+^ Tregs were labeled CFSE (BioLegend, 423801) and cultured in shNC or shGal1 CM containing 50 µM β‐mercaptoethanol, 25 mM HEPES, 1% sodium pyruvate, 2000 U/ml recombinant murine IL‐2 and CD3/CD28 antibody‐coated beads (11456D, Thermo Fisher Scientific). Five days later, cell proliferation of CD4^+^CD25^+^Foxp3^+^ Tregs was assessed by determining the CFSE dilution with flow cytometry.

### Cell Proliferation Assay

The cell counting kit‐8 (CCK‐8, MCE, HY‐K0301) was administrated to assess the proliferation of shNC and shGal1 hepa1‐6 cells according to the manufacturer's instructions. Cells were seeded in 96‐well plates at a density of 2000 cells per well. After adding 10 µl CCK‐8 reagent and incubating at 37 °C for 1 h, the absorbance at a 450 nm wavelength was measured daily.

### Statistical Analysis

All statistical analyses were performed using GraphPad Prism 9 software (La Jolla, CA). Flow cytometry data were analyzed using FlowJo.10 (TreeStar, Ashland, OR). Comparison of data from different groups was achieved utilizing unpaired two‐tailed Student t test or one‐way ANOVA test. Kaplan‐Meier survival curves were plotted and were compared by log‐rank test. *P* < 0.05 was considered statistically significant. (^*^ *P* < 0.05, ^**^ *P* < 0.01, ^***^ *P* < 0.001, ^****^ *P* < 0.0001.)

## Conflict of Interest

The authors declare no conflict of interest.

## Author Contributions

X.Y., J.Q., and L.D. contributed equally to this work. X.Y., J.Q., L.Z., and S.Z. designed and supervised the study. X.Y., J.Q., L.D., R.S., D.G., X.L., M.S., and C.P. performed the experiment. X.Y., J.Q., L.D., Q.W., S.W., and J.L. performed the acquisition and analysis of the data and statistical analysis. All the authors drafted the manuscript. X.Y., J.Q., H.X., S.Y., L.Z., and S.Z. revised the manuscript. All the authors have read and approved the final version of the manuscript.

## Supporting information



Supporting Information

## Data Availability

The data that support the findings of this study are available from the corresponding author upon reasonable request.
