# Supplementary material for: Galectin‐1‐Induced Tumor Associated Macrophages Repress Antitumor Immunity in Hepatocellular Carcinoma Through Recruitment of Tregs
Source: Adv Sci (Weinh). 2025 Jan 24;12(11):2408788. doi: 10.1002/advs.202408788 (PMC11923918; doi:10.1002/advs.202408788)
Supplement: Supplementary file 1 — Supporting Information [file ADVS-12-2408788-s001.docx]

**Supplementary experimental procedures**

**Cell culture, transfection and conditioned medium (CM) collection.**

The hepa1-6 cell line was purchased from the American Type Culture Collection (Manassas, VA) and was cultured in Dulbecco's Modified Eagle Medium (DMEM; Life Technologies, Gaithersburg, MD) supplemented with 10% FBS and 1% penicillin/streptomycin solution (Gibco) at 37°C in a humidified incubator (Thermo Fisher Scientific, USA) with 5% CO2. The cell line was routinely authenticated before use. Gal1 short hairpin RNA (shRNA) lentivirus was synthesized by Hanheng Biotechnology (Shanghai, China) and the sequence was 5ʹ-ACCTGTGCCTACACTTCAA-3ʹ. For CM collection, shGal1 and shNC hepa1-6 cells were seeded at equal numbers in the 10cm dishes. When the cells reached 70% confluence, the medium was changed to RPMI 1640 and incubated for 24 hours to obtain the tumor medium. The CM consisted of a 1:1 mixture of tumor medium and fresh RPM1 1640 medium and was stored at -80°C for further use.

**Mouse tumor models**

C57BL/6J mice were purchased from Hangzhou Ziyuan Experimental Animal Technology (Hangzhou, China). C57BL/6J *Foxp3DTR* mice were purchased from Shanghai Model Organisms (Shanghai, China). To construct a liver orthotopic HCC mouse model, a mixture of DMEM and Basement Membrane Matrix (with the ratio of 1:1, Matrigel, 354248, Corning) containing 5 ×10^6^ hepa1-6 cells (shNC or shGal1) were injected into the left liver lobe of C57BL/6J mice.

**Flow cytometry**

The following antibodies were used for flow cytometry: Zombie NIR™ Fixable Viability Kit（423106), PE/Cyanine7 CD11b (101216), APC F4/80 (123116), Percp-cy5.5 CD45 (103132), rabbit anti-mouse CCL20 antibody (ab9829, Abcam), PE goat anti-rabbit IgG H&L (ab72465, Abcam), Brilliant Violet 650 CD3 (100229), Percp-cy5.5 CD8 (100734), Alexa Fluor 647 Granzyme B (515406), PE Perforin (154306), Brilliant Violet 711 TNF-α (506349), Brilliant Violet 605 IFN-γ (505839), Brilliant Violet 421 LAG-3 (125221), PE/Cyanine7 PD-1 (109110), PE-Cy7 CD45 (103114), Percp-cy5.5 CD8 (100734), APC CD3 (100236), PE NK1.1 (108708), Brilliant Violet 510 CD45 (103138), Brilliant Violet 421 CCR6 (129818), Percp-cy5.5 CD3 (100328), PE/Cyanine7 CD4 (100528), PE Foxp3 (126404) and APC CD25 (102012). Unless otherwise specified, all the antibodies were purchased from Biolegend.

**Immunohistochemistry and immunofluorescence**

EDTA buffer (pH 9.0) was applied to antigen retrieval using the induction cooker for 25 minutes. Tissue sections were then treated with goat serum at 37°C for 40 min and were incubated at 4°C overnight with the following antibodies. For human tissue sections, Galectin-1 (ab138513, Abcam), CD8 (ab101500, Abcam), Foxp3 (ab253297, Abcam) were used. Alexa Fluor 488 and Alexa Fluor 594 secondary antibodies (#4412 and #8890, CST) were used for immunofluorescence. For mouse tissue sections, CD8 (ab217344, Abcam), Foxp3 (ab253297, Abcam) were used.

For cell immunofluorescence, cells were fixed with 4% paraformaldehyde and blocked for 1 hour with blocking buffer containing 4% bovine albumin serum, 0.5% triton X-100 in PBS. Cells were then incubated at 4°C overnight with rabbit anti-mouse P65 (3033s, CST). Goat anti-rabbit Cy3 secondary antibody (AS007, ABclonal) was used. The cytoskeleton was stained with phalloidin (PF00001, Proteintech) for 40 min, and the nucleus was counterstained with DAPI for 10 min. The images were visualized under confocal microscopy (Olympus FV3000, Olympus, Japan).

**Western blot**

Total proteins were extracted using RIPA lysis buffer containing protease inhibitor and phosphatase inhibitor. 10μg total proteins were separated by SDS-PAGE gel electrophoresis and transferred to PVDF membranes. The membranes were then incubated at 4°C overnight with mouse anti-PI3K (4257t, CST), mouse anti-phospho-PI3K (4228t, CST), mouse anti-AKT (4691t, CST), mouse anti-phospho-AKT (4060t, CST), mouse anti-P65 (6956t, CST), mouse anti-phospho-P65 (3033t, CST). HRP-conjugated anti-mouse or anti-rabbit antibodies were used as the secondary antibodies. Protein signals were visualized using the Bio-Rad imager system (Bio-Rad ChemiDoc MP).

**RNA extraction and quantitative RT-PCR**

Total RNA was extracted using the RNA extraction kit () and was reverse transcribed into cDNA with the reverse transcription kit (,Vazyme) according to the manufacturer’s instructions. Real-time quantitative PCR was performed with SYBR qPCR Master Mix (Q711-02, Vazyme). We compared gene expression levels between the groups using the threshold cycle value. Independent experiments were repeated at least three times.

**RNA-sequence and analysis**

BMDMs were co-cultured with tumor cells (shNC or shGal1) in 6-well plates at a ratio of 1:1 for 24 hours. Next, CD11B^+^F4/80^+^ BMDMs were isolated by flow sorting and were used for RNA-sequencing (n = 4). The transcriptome sequencing was conducted by OE Biotech Co., Ltd. (Shanghai, China). The libraries were sequenced on an Ilumina Novaseq 6000 platform. Raw data of fastq format were firstly processed to obtain the clean reads. About 5.88-6.66 G clean reads for each sample were generated and were mapped to the reference genome using HISAT2. FPKM of each gene was calculated using Cufflinks, and the read counts of each gene were obtained by HTSeq-count. Bioinformatic analysis was performed using the OECloud tools at https://cloud.oebiotech.com/task/.

**Table S1**

**Clinical characteristic summary of patients with HCC**

|  | **Gal1^high^ group** | **Gal1^low^ group** | ***P* value** |
| --- | --- | --- | --- |
| age | 53.89 ±10.889 | 57.30 ±9.384 | 0.200 |
| AFP (ng/mL) |  |  | 0.678 |
| >400 | 15 | 13 |  |
| ≤400 | 20 | 14 |  |
| Tumor size (cm) |  |  | 0.036^*^ |
| >5 | 31 | 18 |  |
| ≤5 | 4 | 9 |  |
| TNM stage |  |  | 0.004^**^ |
| I/II | 16 | 22 |  |
| III | 19 | 5 |  |

**Table S2**

**Primer information**

| Gene |  | Primer sequences (5’ to 3’) |
| --- | --- | --- |
| CCL20 | Forward | GTGGGTTTCACAAGACAGATGGC |
|  | Reverse | CCAGTTCTGCTTTGGATCAGCG |
| GAPDH | Forward | CATCACTGCCACCCAGAAGACTG |
|  | Reverse | ATGCCAGTGAGCTTCCCGTTCAG |


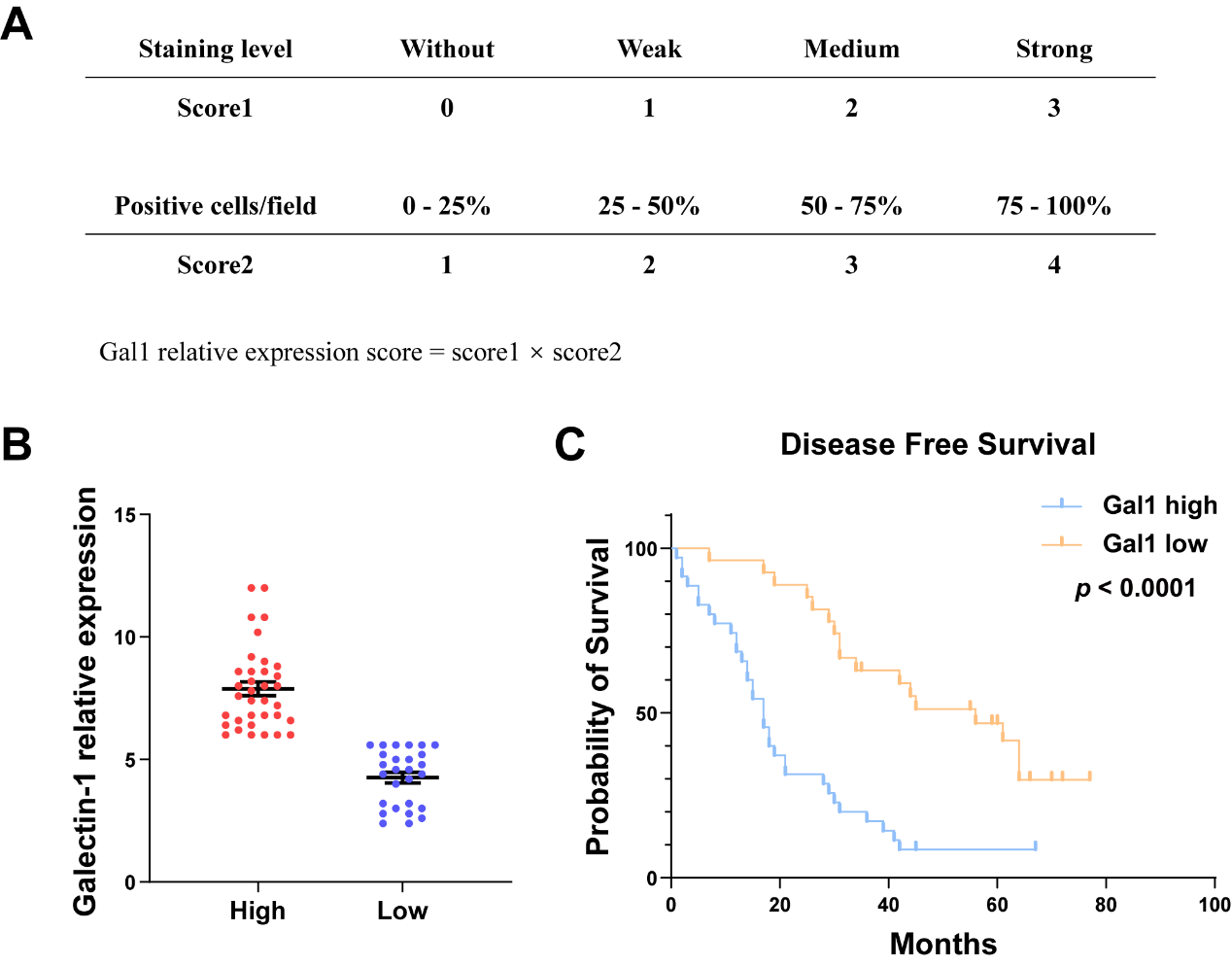


**Fig. S1.** (A) Criteria of immunohistochemical staining score for quantifying the expression of Gal1 in tumor tissues of HCC. (B) Patients with HCC were divided into Gal1 high expression and low expression groups by cut-off value of 6. (C) Kaplan-Meier curve analysis of disease free survival of HCC patients with high Gal1 expression (n = 35) compared with patients with low Gal1 expression (n = 27).


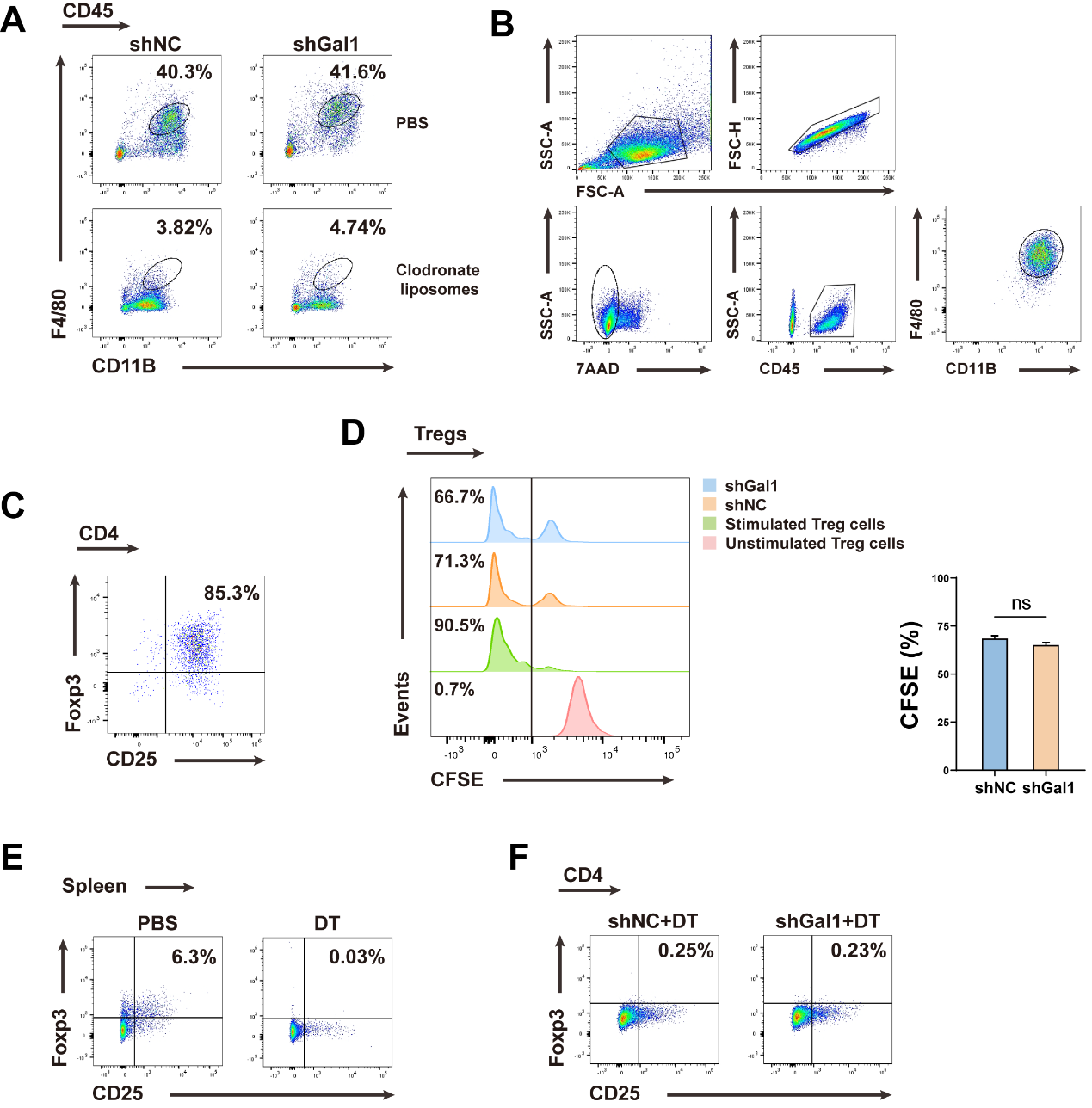


**Fig. S2.** (A) Depletion efficiency of macrophages in the orthotopic HCC models was verified by flow cytometry. (B) Flow cytometry gating strategy for sorting macrophages in the co-culture system. Macrophages were specified as 7AAD^-^CD45^+^F4/80^+^CD11b^+^. (C) The purity of CD3^+^CD4^+^CD25^+^Foxp3^+^ Tregs extracted from mouse spleen was assessed by flow cytometry. (D) CFSE-labeled Tregs were cultured in conditioned medium for 24 hours and cell proliferation was determined by flow cytometry (n = 3). Data were presented as mean ± SEM. (E) *Foxp3DTR* mice were administrated with DT (12.5μg/kg) for Treg depletion, and the depletion efficiency of Tregs in the spleen at day 3 was assessed by flow cytometry. (F) Flow cytometry analysis of the depletion efficiency of Tregs in the orthotopic HCC mouse models. **P* < 0.05; ***P* < 0.01; ****P* < 0.001; *****P* < 0.0001.


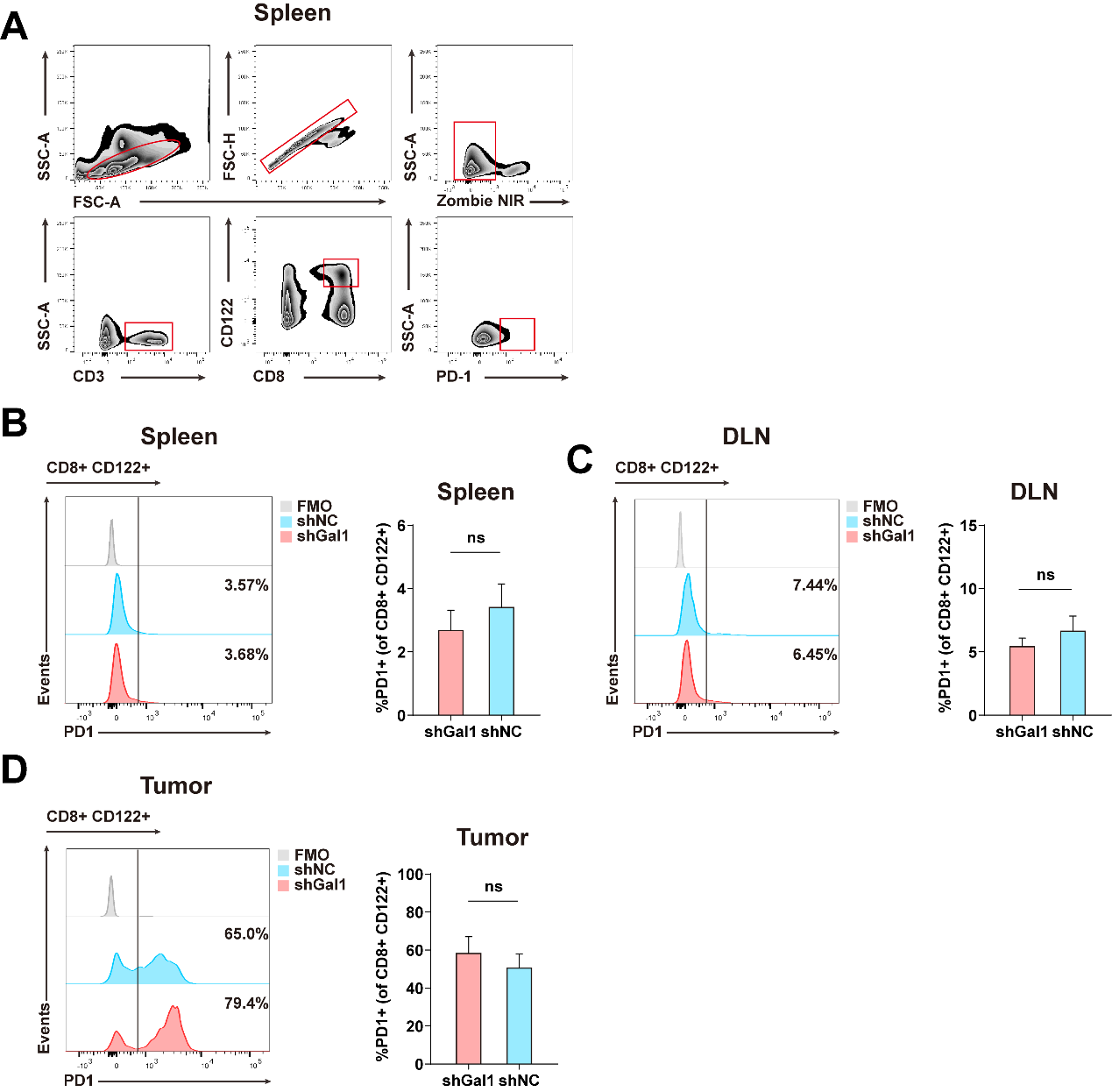


**Fig. S3.** (A) Flow cytometry gating strategy of CD8^+^CD122^+^PD-1^+^ Tregs. (B-D) Flow cytometry analysis of CD8^+^CD122^+^PD-1^+^ Tregs in the mouse spleen (B), tumor draining lymph node (C) and tumor microenvironment (D) in the liver orthotopic HCC mouse models. (n = 5). Data were presented as mean ± SEM. **P* < 0.05; ***P* < 0.01; ****P* < 0.001; *****P* < 0.0001.


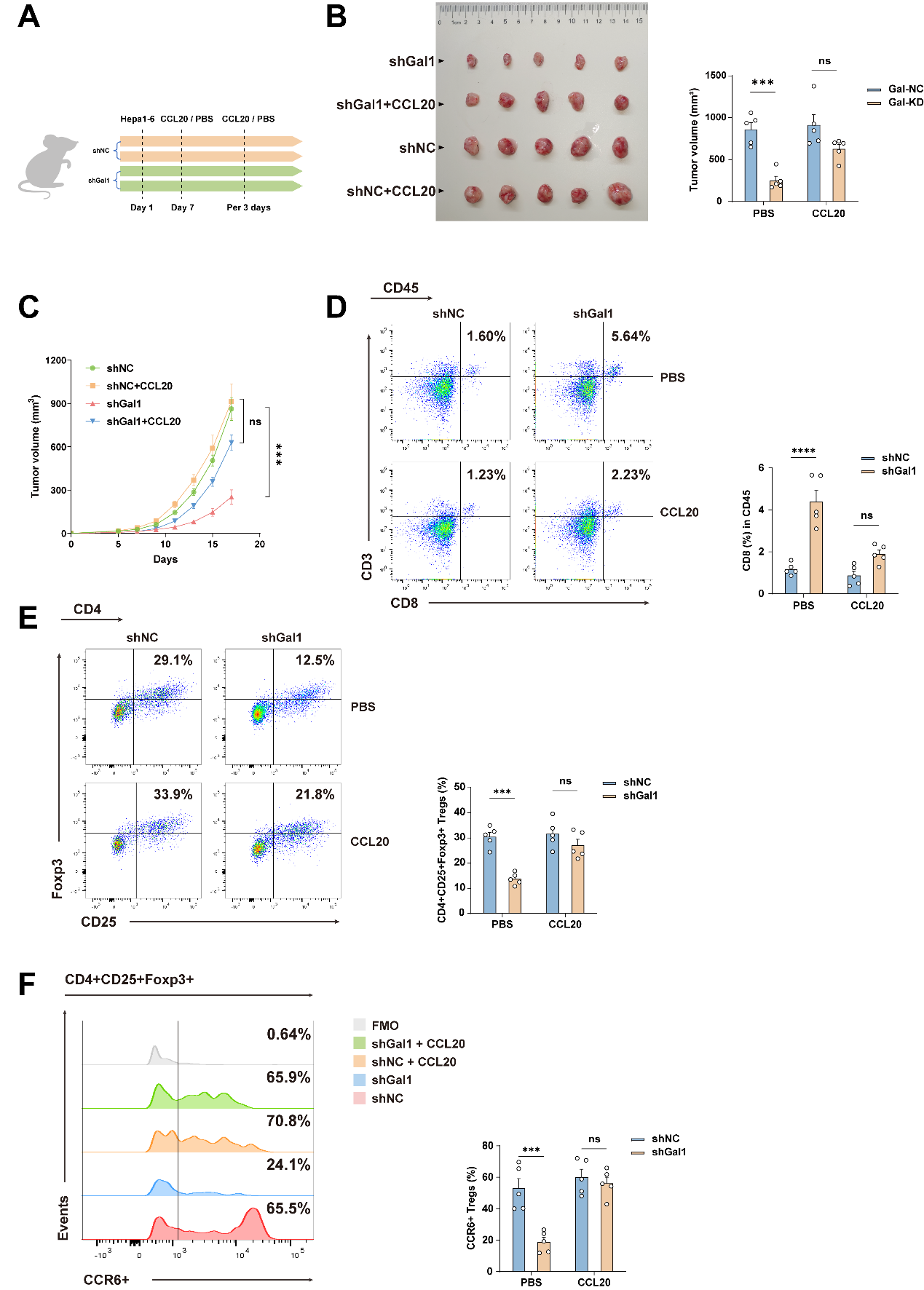


**Fig. S4.** (A) Schematic workflow of CCL20 injecting in subcutaneous HCC mouse models. (B) Image of tumor (left) and tumor volume (right) in the subcutaneous HCC mouse models with or without CCL20 injection (n = 5). (C) Tumor growth curves of subcutaneous HCC mouse models with or without CCL20 injection (n = 5). (D) Flow cytometry analysis of CD8^+^ T cells in subcutaneous HCC mouse models (n = 5). (E) Flow cytometry analysis of CD4^+^CD25^+^Foxp3^+^ Tregs in subcutaneous HCC mouse models (n = 5). (F) Flow cytometry analysis of CD4^+^CD25^+^Foxp3^+^CCR6^+^ Tregs in subcutaneous HCC mouse models (n = 5). Data were presented as mean ± SEM. **P* < 0.05; ***P* < 0.01; ****P* < 0.001; *****P* < 0.0001.


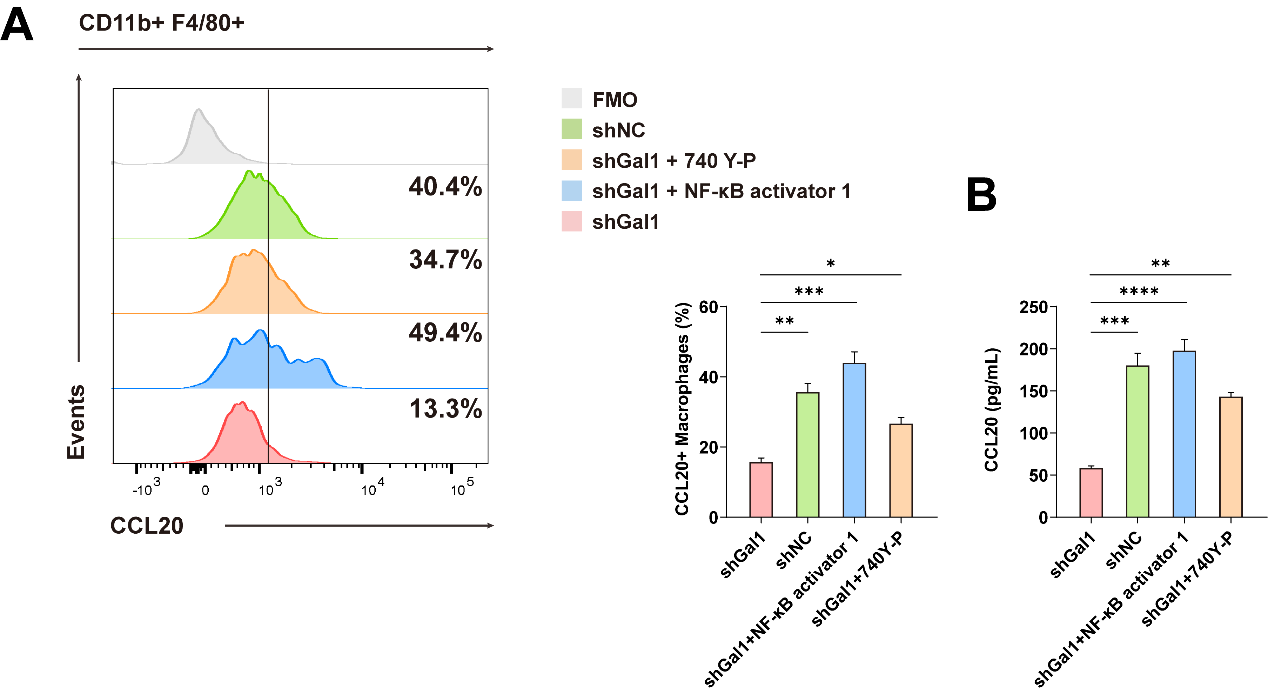


**Fig. S5.** (A) Flow cytometry analysis of CCL20 expression in TAMs (n = 3). (B) ELISA analysis of CCL20 expression in supernatants (n = 3). Data were presented as mean ± SEM. **P* < 0.05; ***P* < 0.01; ****P* < 0.001; *****P* < 0.0001.
